# ABO typing in experimental cynomolgus monkeys using non-invasive methods

**DOI:** 10.1038/srep41274

**Published:** 2017-01-23

**Authors:** Xiaoxiao Wang, Song Chen, Gang Chen

**Affiliations:** 1 Institute of Organ Transplantation, Tongji Hospital, Tongji Medical College, Huazhong University of Science and Technology, Wuhan, 430030, China; 2 Division of Urology and Transplantation, Department of Surgery Sichuan Academy of Medical Sciences and Sichuan Provincial People’s Hospital, Chengdu, 610000, China; 3 Key Laboratory of Organ Transplantation, Ministry of Education, China; 4 Key Laboratory of Organ Transplantation, Ministry of Public Health, China

## Abstract

ABH antigens are not expressed on the red blood cells of monkeys, making it difficult to accurately determine their blood type. In this study, we evaluated the feasibility, convenience, and stability of two non-invasive methods for ABO typing (a reverse gel system assay and a buccal mucosal cell immunofluorescent assay) in cynomolgus monkeys (n = 72). The renal tissue immunofluorescent assay was used to obtain an accurate blood type in the monkeys. Using the reverse gel system assay and preabsorbed serum, we achieved accurate detection of ABO blood groups in 65 of the 72 monkeys but obtained confusing results in the remaining 7. The original immunofluorescent staining of the buccal mucosal smears clearly and correctly identified the ABO blood groups in 50 of the 72 monkeys. After repeated smearing and staining, the ABO group type could be correctly identified in samples from the rest of the monkeys, which were either lacking sufficient buccal mucosal cells or contained impurities. Based on our findings, we recommend the reverse gel system assay as the first choice for primate blood type analysis, and the buccal mucosal cell immunofluorescent assay as a Supplementary Method whenever the reverse gel system assay fails to give a clear result.

Identification of primate ABO blood groups is indispensable in many blood-related research fields, such as organ transplantation and hematology^1^. However, monkeys do not express ABH antigens on their red blood cells (RBCs)^2^, making the accurate determination of blood type difficult. In 2009, we reported for the first time that a reverse gel system assay using preabsorbed serum had been validated as a simple and reliable method for ABO typing of Rhesus and cynomolgus monkeys^3^. However, with the appearance of an increasing number of tests, confusing results are still being obtained in some cynomolgus monkey specimens. Therefore, it is still necessary to find a more effective method for accurate blood type determination in monkeys. In recent years, another non-invasive method with buccal mucosal cell immunohistochemistry has been reported to determine ABO typing of monkeys and obtain good results^4^,^5^. In this study, we have evaluated the feasibility, convenience, and stability of the buccal mucosal cell immunofluorescent assay and investigated its usefulness as a supplement to the reverse gel system assay.

## Results

### ABO typing by renal tissue immunofluorescent staining

**Figure 1:**
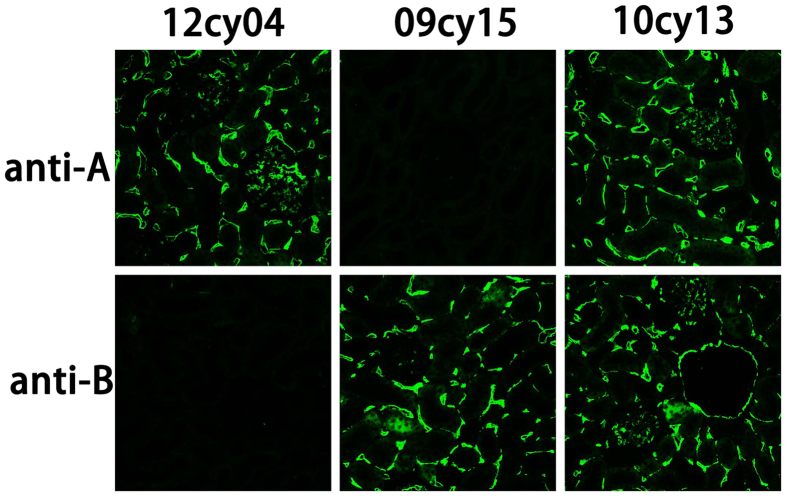
ABO typing by kidney tissue immunofluorescent staining. Kidney sections were stained with mouse anti-human A or B monoclonal antibody, followed by FITC-conjugated secondary antibody (green). Cynomolgus monkeys 12cy04, 09cy15 and 10cy13 showed positive staining for the A, B or A & B antigen in the interstitium and glomerulus of the kidney, and were therefore of blood type A, B, and AB, respectively (magnification ×200).

All renal tissue samples from the monkeys were examined retrospectively with the immunofluorescent staining assay (Fig. 1), yielding an accurate determination of the ABO phenotype of these animals (Table 1). Blood type B was represented by 41.7% of the 72 cynomolgus monkeys typed. There was a relatively equal representation of blood types A and AB (27.8% and 29.2%, respectively). Blood type O was found in only one of these monkeys (approximately 1.3%).

### ABO typing by the reverse gel system assay

**Figure 2:**
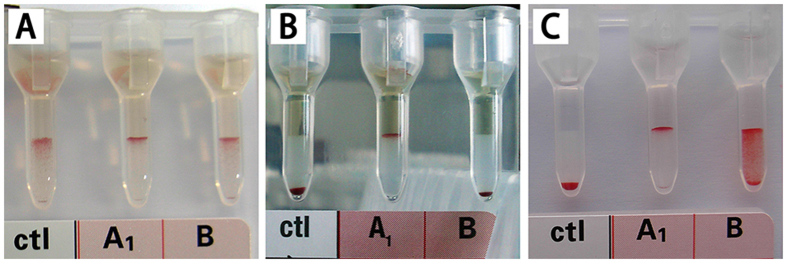
The reverse gel system card used for ABO typing. (**A**) With non-preabsorbed sera, false-positive reactions occurred in the control (“ctl”) tubes, so the results in the “A1” and “B” tubes were not reliable. (**B**) With preabsorbed sera, most results were clear. Typical blood type “B” is shown. (**C**) With preabsorbed sera, some interference still existed in a few samples, but the “ctl” tubes did not show any false-positive reactions.

ABO blood group types were determined by the reverse gel test using preabsorbed serum samples. For the 72 cynomolgus monkeys typed, none of the control tubes showed a false-positive reaction. In 65 of the 72 monkeys, the results from the A1 or B tubes were very clear and definitive, without any interference (Fig. 2B). However, the results from either the A1 or B tubes were still not clear enough in 7 monkeys (approximately 9.7%), even after the repeated adsorption of the serum samples (Fig. 2C). In these 7 monkeys, the determination of ABO group typing was confusing and difficult using this reverse gel system assay (Tables 1 and 2).

### ABO typing by buccal mucosal cell immunofluorescent staining

**Figure 3:**
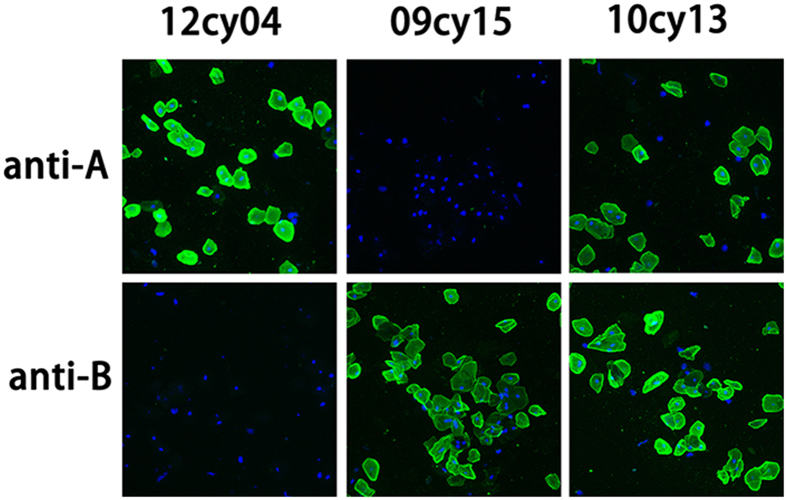
ABO typing by buccal mucosal cell immunofluorescent staining. Samples were first stained with a mouse primary anti-human A or B monoclonal antibody (mAb), followed by FITC-conjugated secondary antibody (green) and DAPI to give blue fluorescent nuclei. Cynomolgus monkey 12cy04 shows positive staining for the A antigen, and is therefore of blood type A (magnification ×200). Monkey 09cy15 shows blood type B, and monkey 09cy15 shows blood type AB (magnification ×200).

The original staining of the buccal mucosal smears clearly and correctly identified 50 of the 72 cynomolgus monkeys, as compared to the results for the renal tissue immunofluorescent staining assay (Tables 1 and 2; Fig. 3). However, the lack of sufficient buccal mucosal cells or the presence of too many impurities in the rest of the 22 stained samples made the determination of ABO group type impossible or difficult. After repeated smearing and staining (16 monkeys needed a single repeat of the test, and 8 monkeys needed two or even more), the ABO group type could be correctly identified in all the monkeys.

## Discussion

Like humans, non-human primates express the ABH specificities of the ABO blood group system. However, these blood antigens are absent or only weakly expressed on the red blood cell (RBC) surface of non-human primates^6^, yet they are widely distributed on the vascular endothelium and epithelial cells and in exocrine secretions^2^,^7^,^8^,^9^. In addition, anti-A and anti-B antibodies may be present in the circulating system of non-human primates. These blood group antibodies can bind to the A/B antigens expressed on the endothelial cells of transplanted organs, resulting in hyperacute or acute humoral injury or rejection similar to that seen in human ABO-incompatible organ transplantation^2^,^7^,^10^. Therefore, accurate detection of primate ABO blood groups is of great significance in relevant research areas and is useful for gaining a more comprehensive understanding of the characteristics of primate blood group systems.

We have reported that the reverse gel system assay with the use of preabsorbed serum samples is a simple and reliable method for ABO typing of both Rhesus and cynomolgus monkeys^3^. However, confusing results, even with repeated absorption treatment, are still occasionally obtained in some cynomolgus monkeys, possibly because of the presence of some unknown non-specific antibodies or other proteins in monkey sera. Thus far, we have not found an effective way to eliminate this interference.

Busch and colleagues have described a method of buccal mucosal cell immunohistochemistry for ABO typing of monkeys^4^. In the present study, we typed 72 cynomolgus monkeys by buccal mucosal cell immunofluorescent staining, and all the final results obtained were consistent with the results obtained from the renal tissue immunofluorescent staining assay. Therefore, the buccal mucosal smear appears to be an accurate method for determining monkey ABO phenotypes. However, in the first smear and staining, insufficient buccal mucosal cells or too many impurities were found in approximately 30% of the samples, making the determination of ABO group type impossible or difficult in the first samples. Therefore, repeated smears and staining were necessary, but the repetition was sufficient to eliminate this interference.

Both of these non-invasive methods have their own advantages and disadvantages. Blood group A/B antibodies present in the serum are determined indirectly by the reverse gel system assay, and thus the results are susceptible to interference by non-specific antibodies and proteins in the serum. In contrast, buccal mucosal cell immunohistochemistry, whose principle resembles that of the immunohistochemistry assay in general, represents a direct determination of histo-blood group A/B antigens present on buccal mucosal cells; therefore, it is more concise and less susceptible to interference by other proteins.

The test specimens of the reverse gel system assay are preabsorbed serum samples, which are advantageous in terms of accessibility and independence from human factors. Although the buccal mucosal cell samples are easily obtained, the test is susceptible to variation in expertise in obtaining the samples and to particles of food being present in the monkey’s mouth.

In the first-round test, the reverse gel system assay had a higher success rate than did the buccal mucosal cell immunofluorescence assay (90.3% vs 69.4%). The reverse gel system assay is a standardized quantitative detection method characterized by simple operation and good repetitiveness, both of which make it easy for a laboratory technician to perform. However, the buccal mucosal cell immunofluorescent staining method is much more complicated and requires a sophisticated professional laboratory technician. In the reverse gel system assay, agglutinated RBCs are separated from non-agglutinated RBCs in microtubes containing dextran acrylamide gel, making the results very clear and definitive, without any interference. The buccal mucosal cell immunohistochemistry assay requires more expertise, with the buccal mucosal cells first needing to be acquired in order to determine their A/B antigen staining.

In summary, as compared to the reverse gel system assay, the buccal mucosal cell immunofluorescence assay is much more complicated and may easily be influenced by artifacts, making it difficult to standardize the process. We recommend that the reverse gel system assay be the first choice in primate blood type analysis, with the buccal mucosal cell immunohistochemistry assay being used as a Supplementary Method whenever the reverse gel system assay fails to give a clear result.

## Materials and Methods

### Animals

All animal studies were approved and performed according to the guidelines of Tongji Medical College ethical committee for animal experimentation. All of the experiments were performed under the guidelines of Tongji animal use regulations and approved by the Institutional Animal Care and Use Committee (IACUC) at the Tongji Medical College, Huazhong University of Science and Technology. Outbred male cynomolgus monkeys (*Macaca fascicularis*) (n = 72), ranging in age from 2 to 5 years, were obtained from the Kunming Institute of Zoology of the Chinese Academy of Sciences (Kunming, China) and Guangzhou Landao Biotechnology Corporation & South China Primates Research Center (Guanzhou, China). The monkeys were housed in the primate facility at the Experimental Animal Center of Tongji Medical College according to the University’s Research Animal Resources guidelines. All animals were originally prepared for other kidney transplant research projects.

### Sample preparations

Buccal mucosal smears were prepared as described previously^4^. In brief, epithelial cells were obtained by repeatedly swabbing the surface of the buccal mucosa with a cotton-tipped applicator and applying the sample to a microscope slide. The slides were dried, fixed in −20 °C acetone (Sigma, St. Louis, MO, USA) for 10 min, and stored at −80 °C until used.

Sera were prepared as we have previously described^3^: Venous blood was collected from the monkeys into separation gel vacuum tubes for preparing serum, and 1 ml of serum collected from each monkey was pre-absorbed on human type O RBCs (Shanghai Blood/Biomedical Co., Ltd, Shanghai, CHN) to eliminate non-specific binding from anti-human heteroagglutinins. Permission to use type O RBCs was obtained from the Tongji Medical College ethical committee.

Normal renal tissues or biopsies were obtained from monkeys during kidney transplant surgery. Tissue and section processing followed general recommendations.

### The reverse gel system assay

The reverse gel agglutination assay was performed using commercially available six-well gel cards (DiaMed-ID Micro Typing System, Cressier FR, Switzerland) as described previously^3^. In brief, a volume of 50 μl of reverse A1 or B reagent RBC suspension and 50 μl of processed serum were pipetted into the appropriate reaction chambers. As a control, reverse O reagent RBC suspension and processed serum were pipetted into the control (“ctl”) chamber in the same proportion (Fig. 1). After incubation and centrifugation, the cards were examined for agglutination.

### Buccal mucosal cell immunofluorescent staining

After air drying and rehydration, the buccal mucosal smears were first incubated in phosphate-buffered saline (PBS, Sigma) containing 10% goat serum for 30 min, then incubated at 4 °C overnight with a diluted primary monoclonal antibody specific for the A or B antigen (Changchun Institute of Biological Products, Jilin, China). After washing with PBS, the smears were incubated with a secondary FITC-conjugated goat anti-mouse antibody (1:100, DAKO, Denmark) in the dark at room temperature for 60 min. After another PBS wash, the smears were analyzed using a fluorescence microscope (Nikon Eclipse TE2000-U, Japan).

### Renal tissue immunofluorescent staining

In order to determine the blood type of the monkeys accurately, A/B antigen was detected by fluorescent staining of formalin-fixed and paraffin-embedded kidney sections retrospectively: After deparaffinization and rehydration, the slides were processed by the same procedure as for the buccal mucosal smears.

## Additional Information

**How to cite this article**: Wang, X. *et al*. ABO typing in experimental cynomolgus monkeys using non-invasive methods. *Sci. Rep.* **7**, 41274; doi: 10.1038/srep41274 (2017).

**Publisher's note:** Springer Nature remains neutral with regard to jurisdictional claims in published maps and institutional affiliations.

## Figures and Tables

**Table 1:** The final results of ABO typing of monkeys.

Blood type	reverse gel system	buccal mucosal cell immunohistochemistry	Staining of monkey kidney tissues (n = 72)*
B	25	30	30 (41.7)
A	18	20	20 (27.8)
AB	21	21	21 (29.2)
O	1	1	1 (1.3)
Difficult to determine	7	0	0
Repetition of determination	0	22 (8**)	0

^*^Values are given as n (%).

^**^Repeat 2 times.

**Table 2:** Outcome of ABO blood typing tests using different methods.

Methods		Cynomolgus monkeys (n = 72)		
		Correct	Incorrect or confusing	Coincidence* (%)
Reverse gel test		65	7	90.3
Buccal mucosal cell immunohistochemistry	By the first staining	50	22	69.4
	After repeated smearing and staining	72	0	100

^*^Compared with the results of kidney tissue immunohistochemical assay.
